# PyNTTTTGT and CpG Immunostimulatory Oligonucleotides: Effect on Granulocyte/Monocyte Colony-Stimulating Factor (GM-CSF) Secretion by Human CD56^+^ (NK and NKT) Cells

**DOI:** 10.1371/journal.pone.0117484

**Published:** 2015-02-23

**Authors:** Juan M. Rodriguez, José Marchicio, Mariela López, Andrea Ziblat, Fernanda Elias, Juan Fló, Ricardo A. López, David Horn, Jorge Zorzopulos, Alejandro D. Montaner

**Affiliations:** 1 Fundación Pablo Cassará, Buenos Aires, Argentina; 2 Immunotech S.A., Buenos Aires, Argentina; 3 David Horn LLC, Doylestown, Pennsylvania, United States of America; 4 Instituto de Ciencia y Tecnología “Dr. Cesar Milstein”, Buenos Aires, Argentina; Beth Israel Deaconess Medical Center, Harvard Medical School, UNITED STATES

## Abstract

CD56^+^ cells have been recognized as being involved in bridging the innate and acquired immune systems. Herein, we assessed the effect of two major classes of immunostimulatory oligonucleotides (ODNs), PyNTTTTGT and CpG, on CD56^+^ cells. Incubation of human peripheral blood mononuclear cells (hPBMC) with some of these ODNs led to secretion of significant amounts of interferon gamma (IFN-γ), tumor necrosis factor alpha (TNF-α) and granulocyte/monocyte colony-stimulating factor (GM-CSF), but only if interleukin 2 (IL2) was present. IMT504, the prototype of the PyNTTTTGT ODN class, was the most active. GM-CSF secretion was very efficient when non-CpG ODNs with high T content and PyNTTTTGT motifs lacking CpGs were used. On the other hand, CpG ODNs and IFNα inhibited this GM-CSF secretion. Selective cell type removal from hPBMC indicated that CD56^+^ cells were responsible for GM-CSF secretion and that plasmacytoid dendritic cells (PDCs) regulate this process. In addition, PyNTTTTGT ODNs inhibited the IFNα secretion induced by CpG ODNs in PDCs by interference with the TLR9 signaling pathway. Since IFNα is essential for CD56^+^ stimulation by CpG ODNs, there is a reciprocal interference of CpG and PyNTTTTGT ODNs when acting on this cell population. This suggests that these synthetic ODNs mimic different natural alarm signals for activation of the immune system.

## Introduction

Highly immunostimulatory oligonucleotides (ODNs) can be classified in two major classes: a) CpG ODNs, which contain at least one CpG motif in their sequence [1] and b) PyNTTTTGT ODNs, which contain at least one motif in their sequence (Py: Pyrimidine; N: Adenine, Cytosine, Thymidine or Guanine; T: Thymidine; G: Guanine) [2]. Some other classes of CpGs, mainly represented by ODNs with a hybrid structure containing motifs of both major classes and therefore a plethora of mixed activities, have been also propose [3–5]. The hallmark of CpG ODNs is their ability to induce secretion of interferon (IFN)-α by plasmacytoid dendritic cells (PDCs) interacting with the endosome associated toll-like receptor (TLR)-9 [6,7]. The structure of the most efficient CpG ODNs for induction of IFNα secretion (CpG ODNs subclass A) fulfills certain sequence requirements (such as being palindromes, and having two poly G ends and phosphorothioate-modified linkages at the 5′ and 3′ ends), which allow them to form nanoparticles [8]. These particles most probably resemble packet viral genomes after elimination of the viral capsid within endocytic vesicles during the first steps of virus cell infection.

Very efficient IFNα inducers such as CpG 2216, the prototype of the CpG ODNs subclass A, are, however, poor inducers of B cell activation [9]. In contrast, PyNTTTTGT ODNs or some hybrid CpG ODNs, such as CpG 2006, the prototype of the CpG ODNs, which has a structure bearing features characteristic of the two major class of immunostimulatory ODNs, are very efficient for B cell activation but null (PyNTTTTGT ODNs) or poor (CpG 2006) IFNα inducers [1,2].

According to their immunostimulatory properties, CpG and PyNTTTTGT ODNs are good adjuvants for vaccines [10–15]. On the other hand, PyNTTTTGT ODNs, but not CpG ODNs, increase the number of mesenchymal stem cells (MSC) precursors in vivo and in vitro [16] and in consequence are good inducers of tissue repair in animals [16–18]. Here, we report another important difference between CpG and PyNTTTGT ODNs: a marked difference in their abilities to stimulate granulocyte/monocyte colony-stimulating factor (GM-CSF) secretion acting on CD56+ cells in human peripheral blood mononuclear cells (hPBMC) in cooperation with IL2. We also describe a reciprocal interference of CpG and PyNTTTTGT ODNs regarding to their specific mode of stimulation of CD56+ cells in hPBMC.

CD56+ cells have been recently recognized as a key link between innate and acquired immune responses [19,20]. On the other hand, T cells play a critical role in this link by secreting IL2, which empowers CD56+ cells to productively respond to a number of stimuli during infection [21,22]. The results here reported agree with this scenario and add new information in particular on the effect exerted by members of the different classes and subclasses of immunostimulatory ODNs on the potential regulatory behavior of human CD56+ cells.

## Material and Methods

### Nucleic acids

Desalted ODNs were purchased from Oligos ETC (Bethel, ME, USA). ODNs were suspended in depyrogenated water, assayed for lipopolysaccharide contamination by using the Limulus test, and kept at -20°C until used. Poly-IC, a synthetic analog of double-stranded RNA, was purchased from Invivogen (San Diego, CA, USA). ODNs had the following sequences: CpG ODN 2216 (Subclass A): 5’-GGgggacgatcgtcGGGGGg-3’; CpG ODN 2006 (Subclass B): 5’-TCGTCGTTTTGTCGTTTTGTCGTT-3’; IMT504 (PyNTTTTGT Class): 5’-TCATCATTTTGTCATTTTGTCATT-3’; IMT022 (non-active ODN): 5’-TGCTGCAAAAGAGCAAAAGAGCAA-3’. Uppercase letters represent PS linkage, whereas lowercase letters represent PO linkage.

### Cell samples

Peripheral blood samples from healthy donors were obtained from 14 buffy coats provided by the Hematological division of the Hospital Alemán (Buenos Aires, Argentina) with the approval of the ethics committee of the Institution ("Comité de Ética Independiente de Investigaciones Clínicas", Hospital Alemán). All participants provided their written informed consent. Data were analyzed anonymously (n: 9 men and n: 5 women; mean age, 46 years). Samples were stored at room temperature prior to gradient separation. Processing was completed within 5 h for all sample specimens. PBMC were isolated by Ficoll-Hypaque density gradient centrifugation (Ficoll-Paque PLUS, GE Healthcare, USA). Briefly, blood samples were diluted 1/2 in RPMI 1640 medium (Gibco, USA) and centrifuged at 1000 g for 40 min at room temperature (22–25°C). After this, the PBMC layer was recovered, washed and cultured in RPMI-1640 medium supplemented with 0.5% (v/v) heat-inactivated fetal bovine serum (FBS), 2.0 mM L-glutamine and 50 mg/ml gentamicin sulfate (Gibco). Unless otherwise stated, 1x10^6^ cells/well (PBMC or PBMC depleted of a specific cell population) were plated in polypropylene U-bottom 96-well microtiter plates in a total volume of 200 μL at 37°C in a 5% CO_2_ humidified atmosphere for 72 h.

FBS was from a single lot previously qualified for low background. Cells were counted using Trypan blue staining and a microscope following an established standard operating procedure (SOP) in the lab. The mean cell viability was 92%. All samples met minimum acceptance criteria of >85% viability and >60% recovery.

### Cell purification or cell depletion

Specific cell populations were purified or depleted from hPBMC by using MACS MicroBeads (Miltenyi Biotec, Bergish Gladbach, Germany) following the manufacturer’s instructions. PDCs, B-lymphocytes, T CD4 lymphocytes, monocytes and Natural Killer (NK) cells were purified from hPBMC by using the separation magnetic system: BDCA-4 cell isolation kit, CD19 B cell isolation kit, CD4 MicroBeads, CD14 MicroBeads and CD56 MicroBeads respectively (Miltenyi Biotec). PBMC specific cell depletion was performed by positive selection. The purity (85–97%) of isolated cells was evaluated by flow cytometry analysis. All samples, assays and data acquisition were according to MIATA guidelines.

### GM-CSF, TNFα, IFNγ and IFNα assay

Culture cells were stimulated with ODNs at 6 μg/ml unless otherwise stated and co-stimulated with 400 IU/ml of human recombinant IL2 (Pablo Cassará Laboratory, Argentina). Supernatants were collected and cytokine levels measured by ELISA. Briefly, 96-well microtiter plates (MaxiSorp Surface Immunoplate, Nunc, Denmark) were coated with the specific antibody (Ab) and blocked with RPMI 1640 medium supplemented with 10% (v/v) heat-inactivated FBS (Gibco). Then, plates were incubated with a biotin-labeled Ab and quantified colorimetrically by peroxidase-conjugated streptavidin (SavHRP Cat. 554066, BD Pharmingen, USA) and 3,3′, 5, 5′-Tetramethylbenzidine substrate. All samples were performed in duplicate.

For the granulocyte-macrophage colony-stimulating factor (GM-CSF) ELISA, purified rat anti-human GM-CSF (Cat. 554502, BD Pharmingen) and biotin rat anti-human GM-CSF (Cat. 554505, BD Pharmingen) were used. Standard curves were carried out with recombinant GM-CSF (GROWGEN-GM300, Gautier Cassará, Buenos Aires, Argentina). The GM-CSF detection limit was 9 pg/ml. For the TNFα ELISA, mouse purified Ab anti-hTNFα (Cat. 551220, BD Pharmingen) and mouse biotin conjugated Ab anti-hTNFα (Cat. 554511, BD Pharmingen) were used. Standard curves were carried out with recombinant hTNFα (Cat. 554618, BD Pharmingen). For the IFNγ ELISA, mouse purified Ab anti-hIFNγ (Cat. 554548, BD Pharmingen) and mouse biotin conjugated Ab anti-hIFNγ (Cat. 554550, BD Pharmingen) were used. Standard curves were carried out with recombinant hIFNγ (Cat. 554616, BD Pharmingen).

IFNα secretion was measured by a sandwich ELISA developed in the laboratory using mouse purified monoclonal Ab anti-hIFNα 2b Clone CA5E6 (Zelltek SRL, Argentina) as capture Ab, rabbit hyper-immune sera anti-hIFNα (Zelltek SRL) and mouse monoclonal Ab anti-rabbit IgG HRP conjugated (Sigma A1949). Standard curves were carried out with recombinant hIFNα (Pablo Cassará Laboratory).

Results of the cytokine assay are presented as the average and standard deviation of three replicates using the same PBMC. Responses of different hPBMC were highly variable in terms of the absolute value reached by a given cytokine. However, the relative efficiency of the different ODNs assayed for induction of cytokine secretion was similar for all the PBMC assayed.

### Inhibition of IFNα secretion

To inhibit IFNα secretion, 1x10^5^ PDCs/well were plated in 96-well microtiter plates at 37°C in a 5% CO_2_ humidified atmosphere for 24 h. Purified cells were stimulated at time zero with ODN CpG 2216 (6 μg/ml) or poly-IC (25 μg/ml) as inducer of INFα secretion and co-cultured with different doses of IMT504 (0, 1, 3, 9 and 27 μg/ml). Supernatants were collected and INFα measured by ELISA as described above.

### IL8 secretion

To evaluate IL8 secretion, 4x10^4^ HEK293 cells stably transfected with the hTLR9 gene (293-XL-hTLR9, Invivogen) were cultured in DMEM glutaMAX (Gibco) without FBS, supplemented with 50 μg/ml Gentamicin sulfate (Gibco), 100 μg/mL Normocin (Cat. ant-nr-1, Invivogen) and the selection antibiotic Blasticidin-S-HCl (Cat. R210–01, Invitrogen, USA) for 18–24 h at 37°C in a 5% CO_2_ humidified atmosphere. Cells were stimulated with regular ODNs or with ODNs heated at 95°C, frozen in ethanol (-80°C), thawed in ice and immediately added to cell cultures. IL8 secretion was evaluated by capture ELISA using purified monoclonal Ab anti-hIL8 (Cat. 554716, BD Pharmingen), biotin conjugated monoclonal Ab anti-hIL8 (Cat. 554718, BD Pharmingen) and recombinant hIL8 standard (Cat. 554609, BD Pharmingen).

### Intracellular staining and flow cytometry analysis

For intracellular staining and flow cytometry analysis, 1x10^6^ PBMC/well were cultured in 96-well round bottom plates in a total volume of 200 μL at 37°C in a 5% CO_2_ humidified atmosphere for 72 h. ODNs and IL2 were added to the medium to reach a final concentration of 6 μg/ml and 400 IU/ml respectively. Five hours before the end of the culture, Brefeldin A (Sigma-Aldrich, USA) was added to the culture to reach a final concentration of 10 μg/ml. After this, cells were harvested and stained with anti-CD3 RPE-Cy5 conjugated (Cat. MCA1711C; Clone S4.1, mouse IgG2a. Serotec, UK) and anti-CD56 FITC conjugated (Cat. MCA2046F; Clon MEM-188, mouse IgG2a, Serotec) to identify cell populations. Fixation and permeabilization medium (Cat. BUF09, Serotec) and anti-GM-CSF PE conjugated (Cat. MCA1689PE; clone BVD2–21CII, rat IgG2a, Serotec) were used for intracellular staining, following the manufacturer’s instructions. All antibodies were incubated in the dark at 4°C for 30 min. Surface stain antibodies were washed with PBS and intracellular anti-GM-CSF antibody was washed using the permeabilization medium. Mouse IgG2a RPE-Cy5 or FITC conjugated was used as isotype control for anti-CD3 RPE-Cy5 or anti-CD56 FITC (Cat. MCA929C and MCA929F, respectively. Serotec). Rat IgG2a PE conjugated was used as isotype control for anti-GM-CSF PE (Cat. MCA1124PE. Serotec). Viability was determined to be more than 80% at 72 h as tested by a supra-vital staining.

The samples were directly acquired after finishing the staining. At least 5x10^4^ events were acquired on a FACScan (Becton Dickinson Immunocytometry Systems, San Jose, CA, USA) equipped with BD CellQuest Pro software version 5.1. The photomultiplier tube voltages were adjusted using unstained cell for all parameters. The mean auto-fluorescence values of unstained cells were adjusted to approximately 100 for all fluorochrome channels. Data were analyzed using the Win MDI Interface Flow Cytometry Application software, version 2.8 (Joseph Trotter, copyright 1993–1998). Gates were set as mentioned in the figure legend. This study was performed using SOPs covering the processing, as well as the staining procedure, data acquisition and gating strategy.

### Statistical analysis

The statistical significance of differences between means was analyzed by two-way ANOVA, followed by a Newman—Keuls test. Differences were considered significant at *p<0.05, **p<0.005 and ***p<0.0005.

## Results

### IMT504 in collaboration with IL2 induces a strong secretion of GM-CSF, IFNγ and TNFα in human PBMC

We have previously reported that, like CpG ODNs, PyNTTTTGT ODNs can activate human B cells and PDCs [2]. We now investigated the induction of the secretion of three key regulatory cytokines, GM-CSF, IFNγ and TNFα, mediated by representative members of the PyNTTTTGT and CpG classes of immunostimulatory ODNs acting on hPBMC.

**Fig 1:**
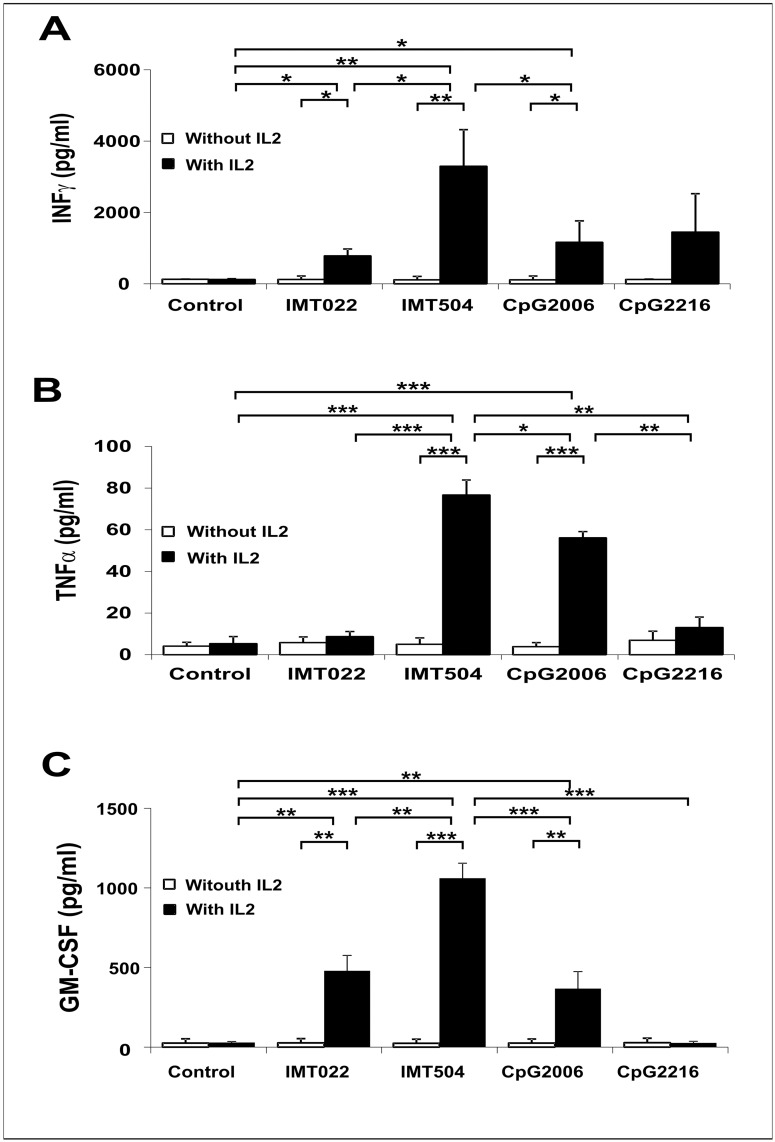
Secretion of IFNγ, TNFα and GM-CSF induced by immunostimulatory ODNs. Effect of IL2 on the secretion of (A) IFNγ, (B) TNFα and (C) GM-CSF on hPBMC. IMT504 is the prototype of the PyNTTTTGT ODN class, CpG 2006 is the prototype of the B subclass of the CpG ODN class, CpG 2216 is the prototype of the A subclass of the CpG ODN class and IMT022 is an ODN with very low immunostimulatory capacity according to our previous studies [2]. ODNs and IL2 were added to the medium to reach a final concentration of 6 μg/ml and 400 IU/ml respectively. Data are shown as mean +SD of duplicates from the same PBMC and are representative of three independent experiments. Two-way ANOVA interaction, p<0.001. Newman—Keuls test: *p<0.05, **p<0.005, ***p<0.0005.

In addition to the presence of an immunostimulatory ODN, IL2 is a requirement for strong secretion of these cytokines by hPBMC (Fig. 1). Members of both classes were able to induce IFNγ and TNFα secretion (Fig. 1A and B) being IMT504 (the prototype of the PyNTTTTGT class) the most efficient among the ODNs assayed. In contrast, GM-CSF secretion was very efficient only upon incubation with IMT504 and comparatively poor or null upon incubation with two representative CpG ODNs, CpG 2006 and CpG 2216 (Fig. 1C). It is also worth noting that IMT022, an ODN with very poor activity according to several markers for immunostimulation [2], had in this case a significant activity as an inducer of GM-CSF secretion.

The results show that PyNTTTTGT ODNs are efficient inducers of the secretion of IFNγ, TNFα and GM-CSF in collaboration with IL2 and that CpG ODNs can induce secretion of significant amounts of IFNγ and TNFα but are comparatively poor or null inducers of GM-CSF secretion. The fact that CpG 2006, which is a relative poor inducer of IFNα secretion [23], was able to induce the secretion of a measurable amount of GM-CSF and the fact that CpG 2216, which is a very good inducer of IFNα [6], was unable to induce any significant secretion of GM-CSF suggest that IFNα may act as an inhibitor of GM-CSF secretion. Evidence supporting this hypothesis is presented.

### Optimal ODN structure for induction of GM-CSF secretion in human PBMC

**Fig 2:**
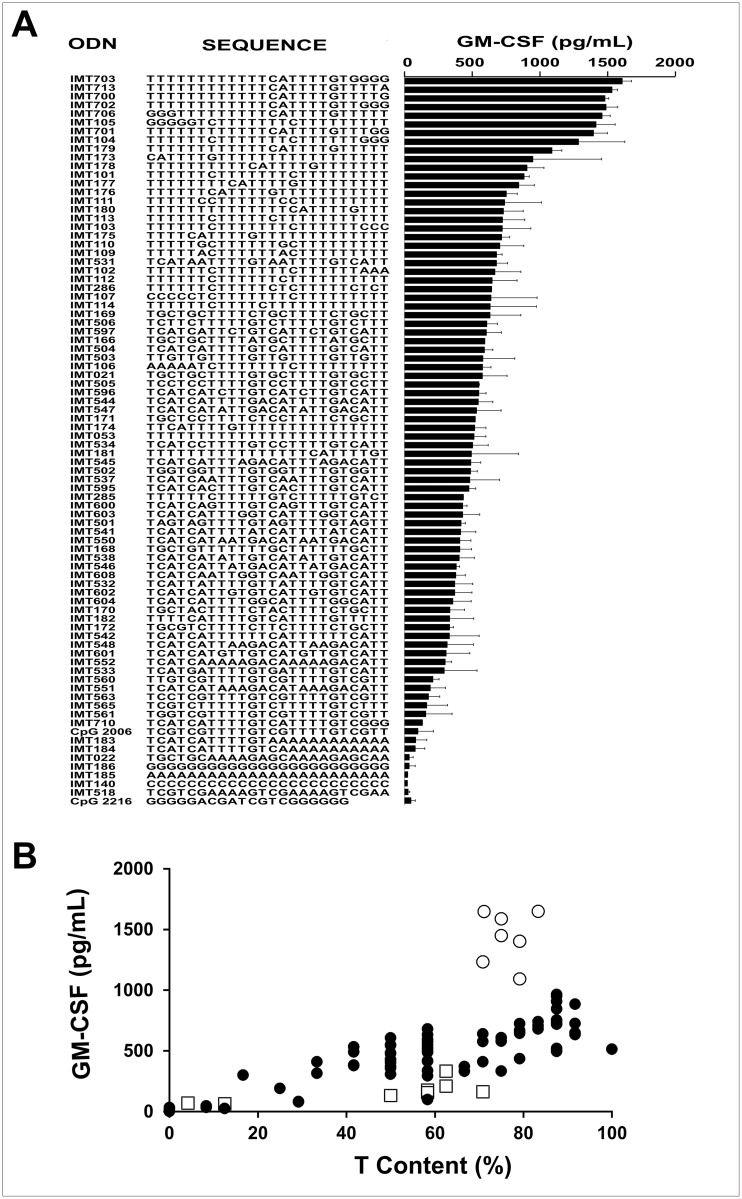
Immunostimulatory ODN sequences and GM-CSF secretion. Induction of GM-CSF by ODNs on hPBMC in the presence of IL2. (A) ODN sequence vs. activity and (B) T content (%) vs. activity. Empty circles represent ODNs with unusual high activity. All these ODNs contain high T content and at least one PyNTTTTGT group (IMT700, IMT703, IMT702, IMT706, IMT701, IMT105, IMT104). Empty squares represent all the CpG ODNs assayed which, in addition to the CpG motif, have a high T content (IMT172, IMT560, IMT563, IMT565, IMT561, IMT710, CpG 2006). ODNs and IL2 were added to the medium to reach a final concentration of 6 μg/ml and 400 IU/ml respectively. Data are shown as mean +SD of duplicates from five independent experiments. Two-way ANOVA interaction, p<0.001. Newman—Keuls test: *p<0.05, **p<0.005, ***p<0.0005.

To confirm that efficient induction of GM-CSF secretion in PBMC was a general property of immunostimulatory ODNs of the PyNTTTTGT class, different ODNs were assayed. Fig. 2A shows the sequences of the ODNs studied organized according to their activity and Fig. 2B shows a plot of activity vs. the T content of each ODN. In general, there was a good correspondence between the T content and activity. However, two groups of ODNs departed from linearity. The first of these two groups (open squares in Fig. 2B) consisted of ODNs (mainly CpG ODNs) with low activity in spite of their high T content. Furthermore, none of the ODNs assayed containing CpG motifs showed good activity as GM-CSF inducers. This result confirms that CpG motifs are detrimental for induction of GM-CSF secretion in hPBMC. The second group (open circles in Fig. 2B) consisted of ODNs which had activity clearly superior to that corresponding to their T content. All members of this group contained a CATTTTGT motif located from the middle of the ODN sequence towards its 3′ end.

In conclusion, high T content and the presence of a CATTTTGT motif located from the middle of the sequence towards its 3′ are important features that an ODN should have to be a very good inducer of GM-CSF secretion in hPBMC in collaboration with IL2.

### IFNα and CpG ODNs strongly inhibit the secretion of GM-CSF induced by IMT504 in human PBMC

**Fig 3:**
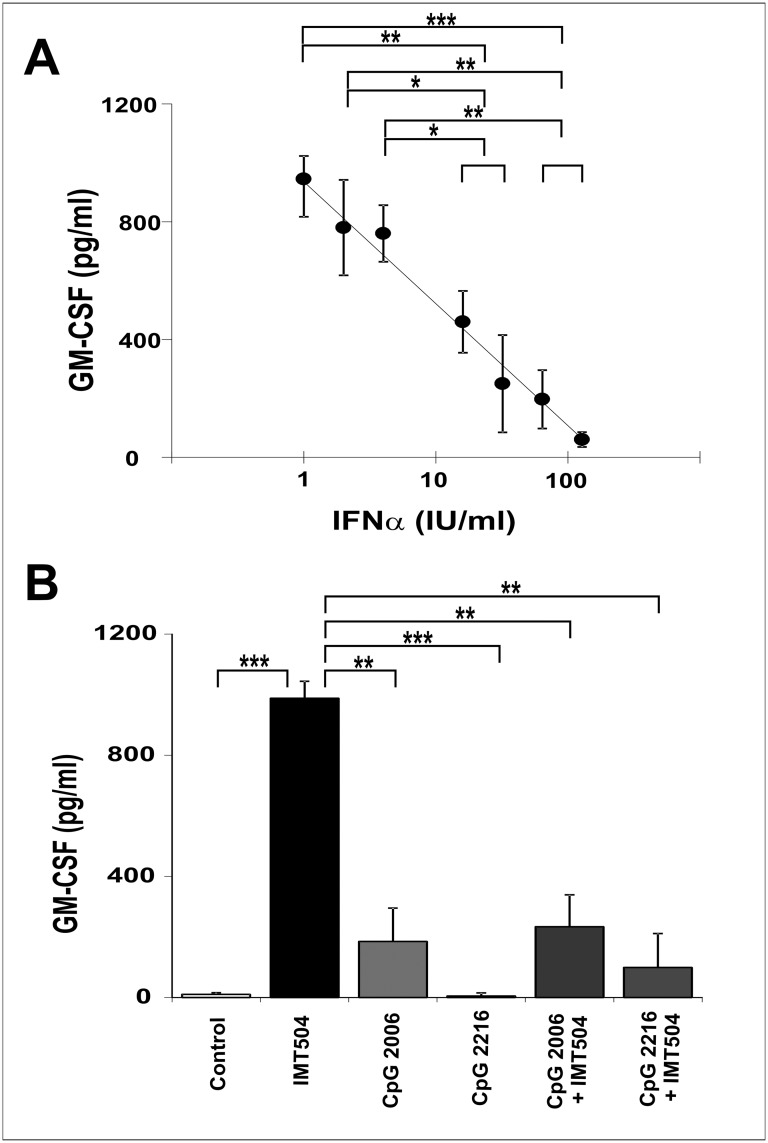
Effect of IFNα and CpG ODNs on GM-CSF secretion induced by IMT504 in hPBMC. Addition of (A) IFNα or (B) CpG ODNs to the hPBMC culture in the presence of IL2. ODNs and IL2 were added to the medium to reach a final concentration of 6 μg/ml and 400 IU/ml, respectively. IFNα was added to the medium to reach the concentration indicated for each point in the figure. Data are shown as mean +SD of duplicates from three independent experiments. Two-way ANOVA interaction, p<0.001. Newman—Keuls test: *p<0.05, **p<0.005, ***p<0.0005.

To study if the secretion of IFNα induced by CpG ODNs was responsible of the low activity displayed by these ODNs during assays of GM-CSF secretion in human PBMC, cells were incubate with different amounts of IFNα during the assay. The addition of this cytokine resulted in a dose-dependent inhibition of the secretion of GM-CSF induced by IMT504 (Fig. 3A). On the other hand, CpG ODNs not only were inefficient in inducing GM-CSF secretion by hPBMC but also inhibited the GM-CSF secretion induced by IMT504 (Fig. 3B). These results strongly suggest that induction of IFNα secretion by CpG ODNs acting on PDCs is involved in both their poor performance as inducers of GM-CSF secretion and their inhibitory effect on GM-CSF secretion induced by IMT504 in hPBMC.

### Selective elimination of PDCs from human PBMC results in highly efficient induction of GM-CSF secretion by all the ODNs assayed regardless of their sequence

**Fig 4:**
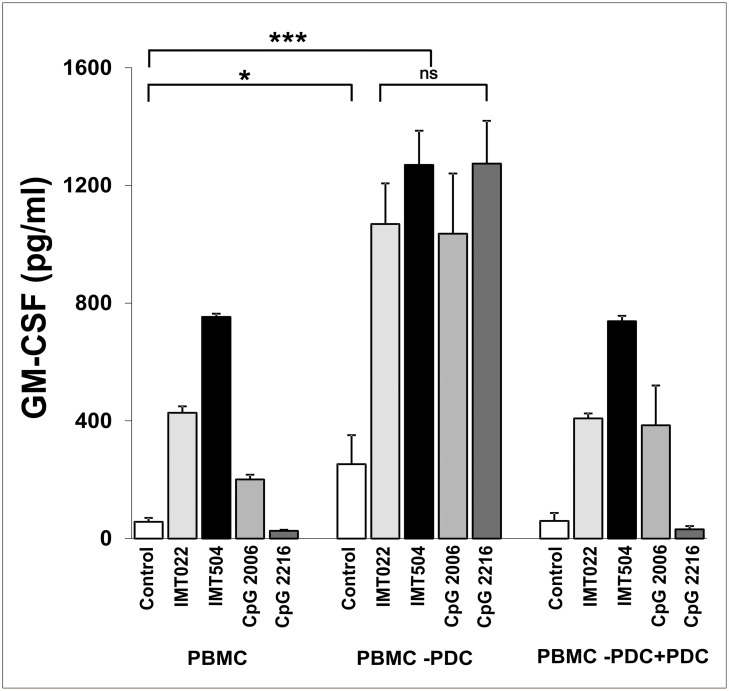
Effect of PDC cell depletion from hPBMC on GM-CSF secretion induced by immunostimulatory ODNs. GM-CSF secretion in the presence of IL2. PDC depletion (-PDC) was performed by positive selection using the MACS MicroBeads system (Miltenyi Biotec, Bergish Gladbach, Germany) and confirmed by flow cytometry. Human peripheral blood mononuclear cells (PBMC) were reconstituted by addition of purified PDCs (+PDC) to reach the same proportion of these cells present in the original PBMC. ODNs and IL2 were added to the medium to reach a final concentration of 6 μg/ml and 400 IU/ml, respectively. Data are shown as mean +SD of duplicates from three independent experiments. Two-way ANOVA interaction, p<0.001. Newman—Keuls test: *p<0.05, **p<0.005, ***p<0.0005.

PDCs are the main IFNα producers among hPBMC [24]. Therefore, to investigate their role in GM-CSF secretion induced by IMT504, we selectively eliminated PDCs from hPBMC. PBMC deprived of PDCs were able to secrete high amounts of GM-CSF in the presence of IL2 even in the absence of immunostimulatory ODNs (Fig. 4). However, secretion of GM-CSF in the presence of ODNs was clearly higher and surprisingly independent of the ODN sequence. Reintroduction of PDCs in the deprived PBMC resulted in re-establishment of the normal pattern of response of PBMC to the different ODNs. Taken into account these and other above-described results, we can conclude that PDCs inhibit GM-CSF secretion induced by ODNs by a mechanism, which most likely involves IFNα secretion. However, we cannot completely rule out some cell-to-cell contact effect also contributing to the inhibition process.

### CD56^+^ are the main GM-CSF-secreting cells during incubation of human PBMC with IL2 plus IMT504

**Fig 5:**
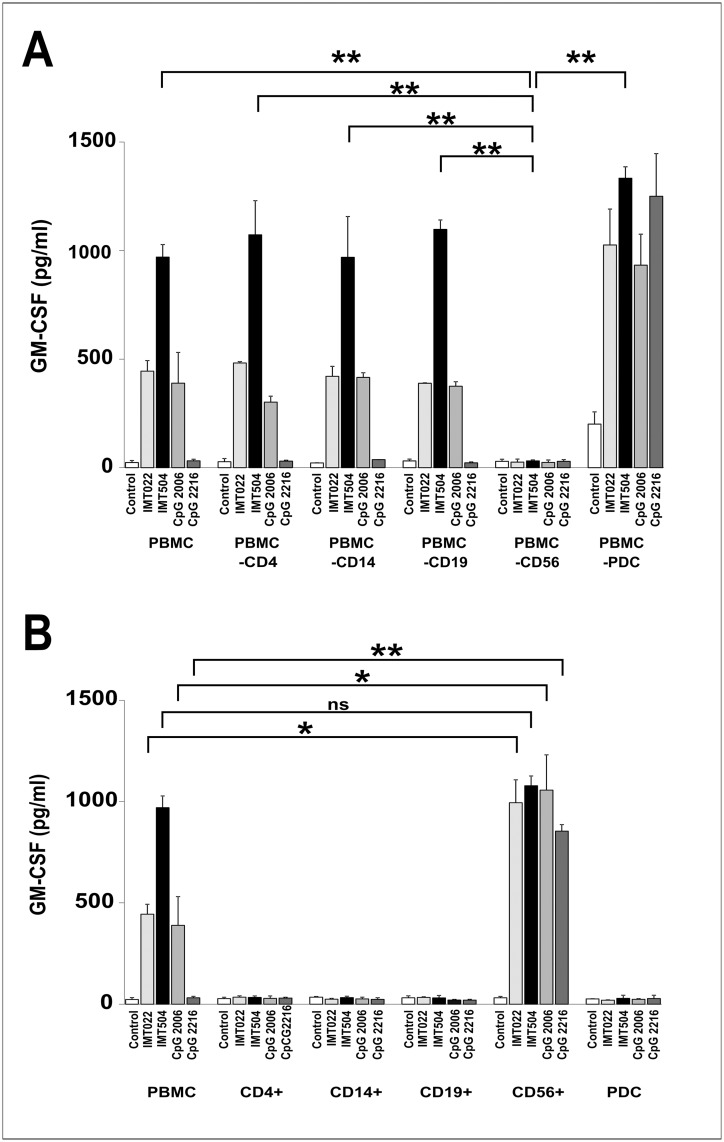
Cell populations from hPBMC and GM-CSF secretion induced by immunostimulatory ODNs. (A) hPBMC depleted of certain cell populations and (B) cell populations isolated from PBMC. Cell depletion/isolation was performed using the MACS MicroBeads (Miltenyi Biotec, Bergish Gladbach, Germany) system and confirmed by flow cytometry. ODNs and IL2 were added to the medium to reach a final concentration of 6 μg/ml and 400 IU/ml, respectively. Data are shown as mean +SD of duplicates from the same PBMC and are representative of three independent experiments. Two-way ANOVA interaction, p<0.001. Newman—Keuls test: *p<0.05, **p<0.005, ***p<0.0005.

**Fig 6:**
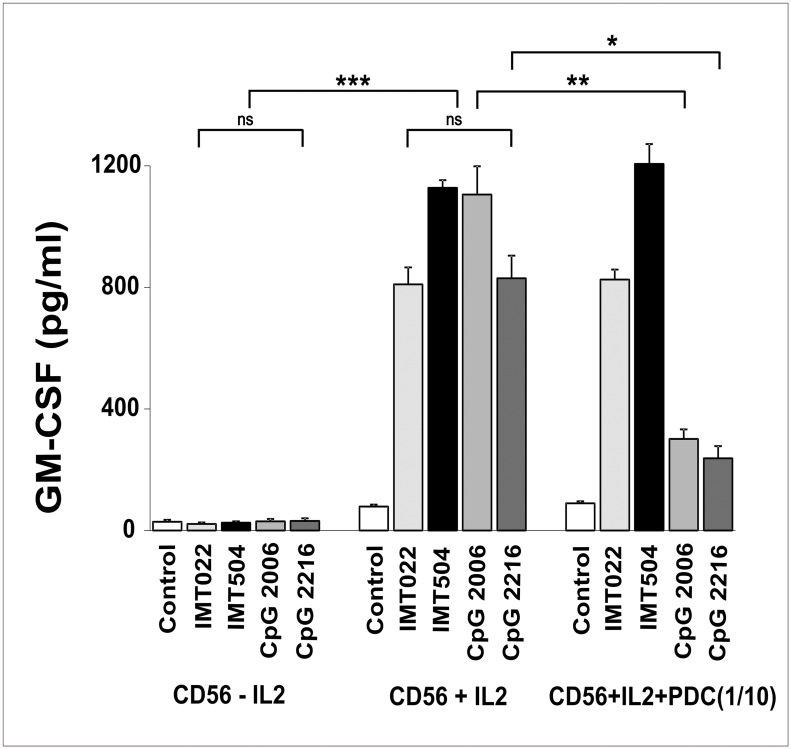
Effect of IL2 and PDC on the ability of immunostimulatory ODNs to stimulate GM-CSF. GM-CSF secretion on purified CD56^+^ cells from hPBMC. CD56^+^ cells and PDCs were purified using the MACS MicroBeads system (Miltenyi Biotec, Bergish Gladbach, Germany) and confirmed by flow cytometry. PDCs were added to purified CD56^+^ cells in a 1 to 10 ratio. ODNs and IL2 were added to the medium to reach a final concentration of 6 μg/ml and 400 IU/ml respectively. Data are shown as mean +SD of duplicates from three independent experiments. Two-way ANOVA interaction, p<0.001. Newman—Keuls test: *p<0.05, **p<0.005, ***p<0.0005.

Several cell types are able to secrete GM-CSF under appropriate stimuli [25]. To investigate which cells were responsible for GM-CSF secretion induced in hPBMC by IMT504 in collaboration with IL2, major cell types were selectively eliminated from PBMC, and GM-CSF secretion analyzed using the residual cell mix. IMT504-induced GM-CSF secretion was positive in all cases except when CD56^+^ cells were absent (Fig. 5A). Moreover, purified cells representing main PBMC cell types were unable to secrete GM-CSF under stimulation with IMT504 plus IL2, except for CD56^+^ cells (Fig. 5B). The GM-CSF secretion pattern for purified CD56^+^ cells was similar to that observed using PBMC deprived of PDCs in which every ODN assayed was a good inducer regardless of its sequence. Furthermore, in this case, both ODN and IL2 were necessary to induce the strongest levels of GM-CSF secretion (Fig. 6). On the other hand, mixing purified CD56^+^ cells with purified PDCs resulted in restoration of the normal pattern of response to specific ODNs, as observed in PBMC. Recently, activation of highly purified mouse and human CD4^+^T cells in the presence of anti-CD3 antibody by ODNs regardless of their sequence has also been reported [26]. This suggests that ODNs may possess enough information in their general structure to stimulate cytokine secretion and that regulation of this secretion strongly depends on the specific cellular environment. Therefore, the results obtained with purified cell populations should be interpreted with caution.

**Fig 7:**
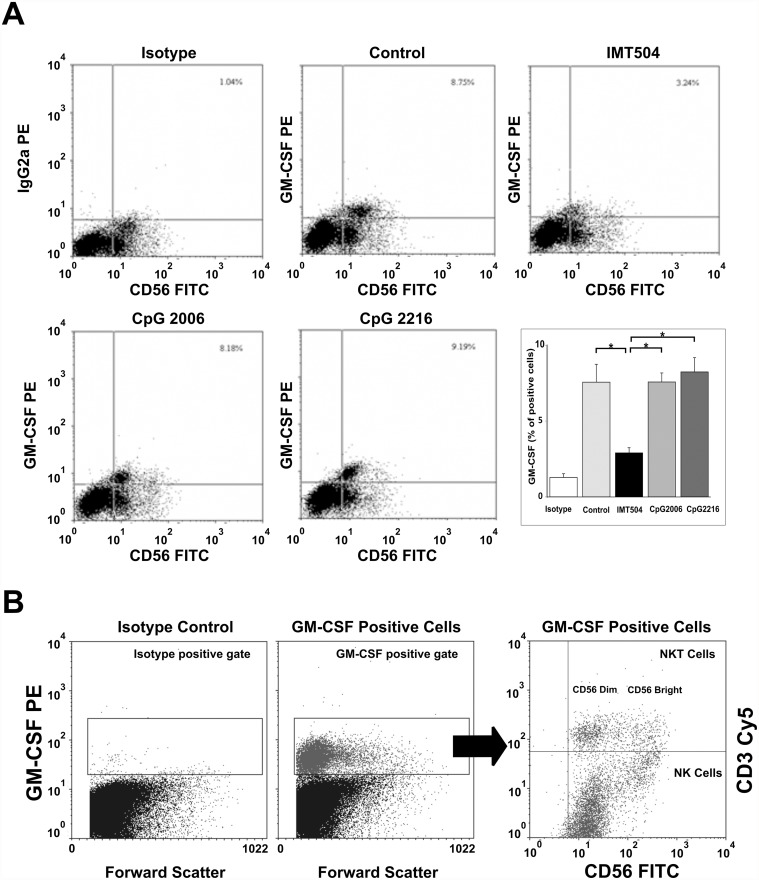
Intracellular GM-CSF staining on CD56^+^ cells from hPBMC. (A) In the presence of the indicated ODN plus IL2: GM-CSF content in the cell culture without ODN stimulation was used as a control. Data are shown as mean +SD and are representative of three similar experiments. A specific response was defined as a response where the percentage of GM-CSF in CD56^+^ lymphocytes was at least 2-fold below the background observed in the medium control. The asterisk indicates statistically significant differences (p<0.05). (B) In the presence of IL2 in both CD3^+^ (NKT) and CD3^-^ (NK) CD56^+^ dim or bright populations: ODNs and/or IL2 were added to the medium to reach a final concentration of 6 μg/ml and 400 IU/ml respectively. Dot plots are representative of three similar experiments.

Flow cytometry analysis showed that CD56^+^ cells are the main cell subpopulation significantly loaded with GM-CSF in hPBMC (Fig. 7A). Upon treatment with IMT504 but not with CpG 2216, the GM-CSF content strongly decreased in the CD56^+^ cell population. This decrease in the GM-CSF cell content was coincident with an increase in the GM-CSF released into the medium as shown in Fig. 1C. As expected, cells treated with IL2 and CpG 2006 showed a modest decrease in their GM-CSF content since, as shown in Fig. 1C, secretion in the presence of this ODN is low. On the other hand, both CD56^+^CD3^-^ bright or dim (NK) cells and CD56+CD3+ (alpha beta and gamma delta NKT cells) were loaded with GM-CSF in the presence of IL2 (Fig. 7B). Taken together, these results indicate that CD56^+^ cells are responsible for the bulk of GM-CSF secretion induced in PBMC by IL2 plus PyNTTTTGT ODNs and that GM-CSF is efficiently synthesized in the cells incubated with IL2 but is continuously secreted by ODN stimulation. Therefore, intracellular accumulation is hindered. Brefeldin A treatment during the last five hours of the 72 h incubation period is most likely responsible for the low amount of intracellular GM-CSF accumulated in cells incubated with IMT504 (Fig. 7A). In contrast, even though CpG ODNs do not suppress the GM-CSF synthesis induced by IL2, since cells incubated with these ODNs are loaded with the cytokine, they are not at all efficient inducers of the cytokine secretion as previously shown in Fig. 1C.

### IMT504 is an inhibitor of the IFNα secretion induced in PDCs by CpG ODNs

**Fig 8:**
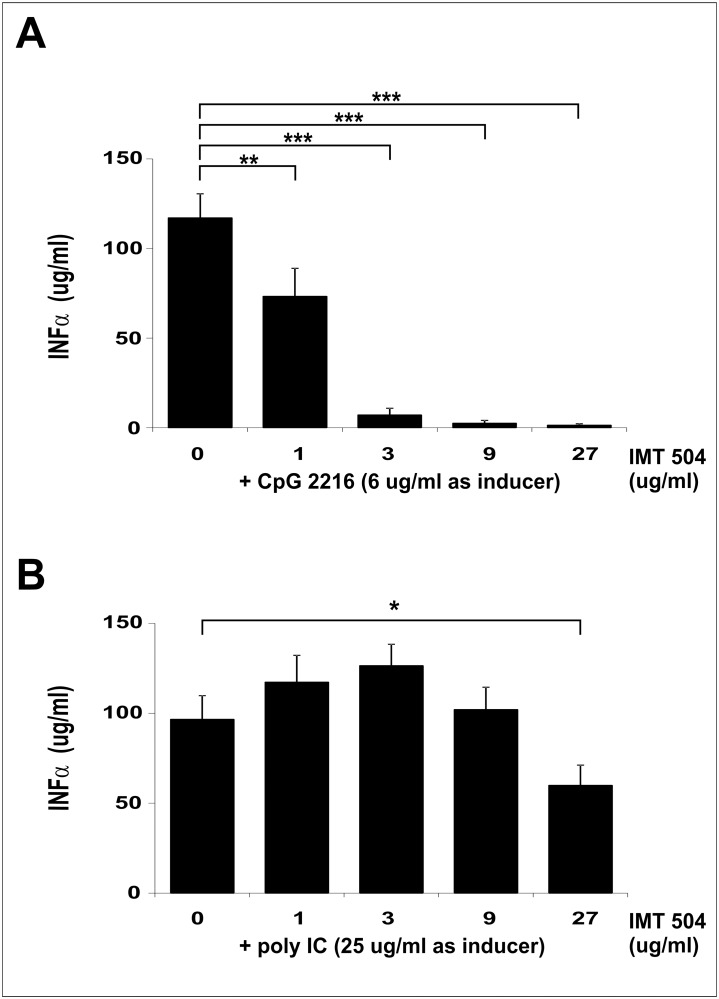
Effect of IMT504 on the IFNα secretion induced by CpG or Poly-IC ODNs on hPDCs. (A) CpG 2216 ODN; (B) Poly-IC on human PDCs. Data are shown as mean +SD of duplicates from three independent experiments. Two-way ANOVA interaction, p<0.001. Newman—Keuls test: *p<0.05, **p<0.005, ***p<0.0005.

**Fig 9:**
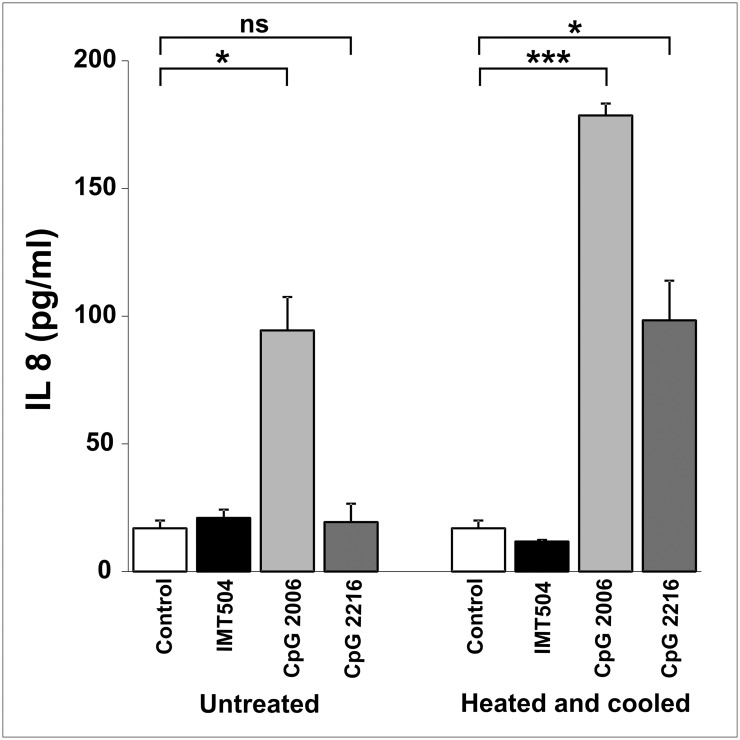
IL8 secretion induced by heated and cooled immunostimulatory ODNs on HEK293 cells stably transfected with the hTLR9 gene. Differences in IL8 secretion after incubation with immunostimulatory ODNs with or without previous heating (95°C, 10 min), rapid freezing in ethanol (-80°C) and thawing on ice. Data are shown as mean +SD of duplicates from three independent experiments. Two-way ANOVA interaction, p<0.001. Newman—Keuls test: *p<0.05, **p<0.005, ***p<0.0005.

We have previously reported that members of the PyNTTTTGT class of immunostimulatory ODNs are unable to induce significant secretion of IFNα acting on PDCs but able to induce the expression of several surface activation markers in these cells [2]. In addition, we are now reporting that IMT504 is an inhibitor of the IFNα secretion induced in PDCs by CpG ODNs. IMT504 inhibited the IFNα secretion induced by the highly efficient CpG 2216 ODN in a dose-dependent manner (Fig. 8). In contrast, we observed only a modest inhibition of the TLR3-dependent IFNα secretion induced by polyinosinic-polycytidylic acid (poly-IC) at high IMT504 concentrations. This result suggests that the inhibition by IMT504 of the IFNα secretion induced by CpG ODNs in PDCs is due to a specific interference in the TLR9 signaling pathway. To test this hypothesis, we used TLR9-expressing recombinant HEK293 cells that produce IL8 upon ligation of the TLR9 receptor. In agreement with previous findings [7], CpG 2006 ODN was able to activate this system (Fig. 9). In contrast, IMT504 was inactive. However, to our surprise, the highly efficient IFNα inducer CpG 2216 was also inactive. This anomaly was eliminated by heating and cooling the ODNs. The simplest explanation for these results is that high-order structures are formed during heating and cooling and that these structures are the effective activators of the TLR9 signaling, as previously reported [8]. The activity of CpG 2006 was also improved by heating and cooling. It is interesting that this CpG ODN is very efficient in stimulating TLR9 signaling in the TLR9-HEK293 system in spite of being a poor inducer of IFNα acting on PDCs. On the other hand, heating and cooling was not effective for IMT504, a result that strongly suggests that PyNTTTTGT ODN activation of the human immune system is most likely TLR9 independent.

Co-incubation of ODN 2006 and IMT504 resulted in inhibition of the IL8 production induced by this CpG ODN in the TLR9-HEK293 system. Thus, this result also suggests that the observed inhibition by IMT504 of the IFNα secretion induced by the CpG-A ODN 2216 in PDCs is due to interference in the TLR9 signaling pathway. In contrast, it has been recently reported that, in addition of being poor inducers of IFNα secretion, CpG-B ODNs are able to inhibit both TLR-dependent and-independent induction of IFNα by CpG-A ODNs, in mouse and human cells at the level of transcription regulation, affecting all the IFN-inducing pathways [27]. This may explain why CpG 2006, which belongs to the CpG-B subclass, is a poor inducer of IFNα secretion in PDCs even though it is a very good activator of TLR9-dependent IL8 secretion in the TLR9-HEK293 system.

## Discussion

**Fig 10:**
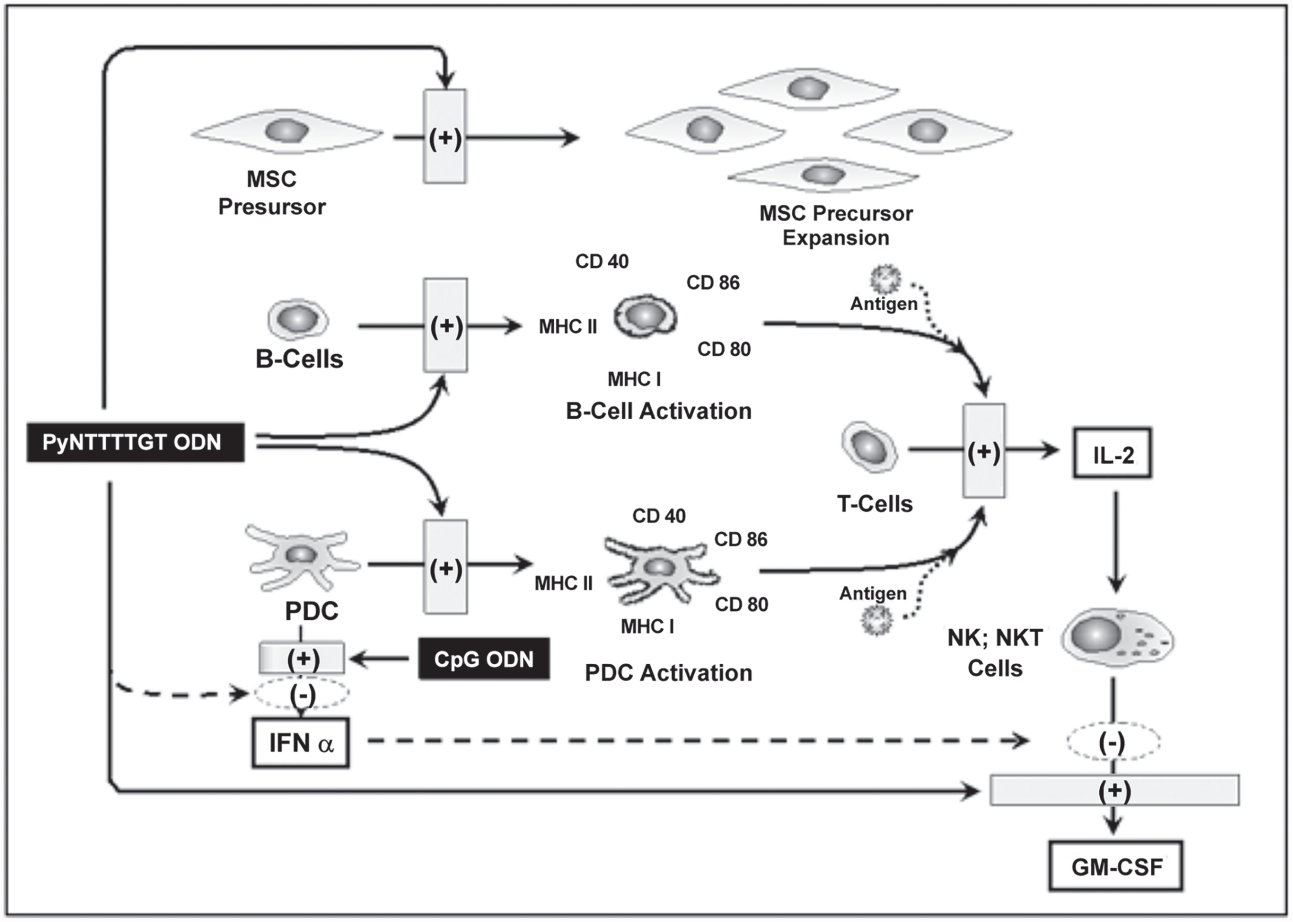
Schematic interpretation of the biological action of PyNTTTTGT ODNs.

When acting on human cells, CpG and PyNTTTGT immunostimulatory ODNs have a number of characteristics in common, but also remarkable differences. Among the most important differences are the ability of PyNTTTTGT but not of CpG ODNs to stimulate proliferation of MSC progenitor cells [16] and the ability of CpG ODNs but not of PyNTTTTGT ODNs to induce IFNα secretion [2]. Here, we described a third significant difference that is stimulation of GM-CSF secretion in hPBMC in collaboration with IL2, which is efficient if the inducer is a PyNTTTTGT ODN and null or poor if the inducer is a CpG ODN. CD56^+^ cells are the main cells responsible for GM-CSF secretion induced by PyNTTTTGT ODNs in hPBMC and, according to flow cytometry analysis, they are loaded with GM-CSF after incubation with IL2, a well-known inducer of the GM-CSF gene transcription in NK cells [28]. However, to be very efficient, GM-CSF secretion needs additional stimulus of an ODN with specific structural features as shown in Fig. 2. PDCs are key players in the regulation of GM-CSF secretion induced by PyNTTTTGT ODNs, and removal of these cells from hPBMC results in loss of the ODN specificity since, in these conditions, PyNTTTTGT ODNs and CpG ODNs are able to stimulate a very strong secretion of GM-CSF in collaboration with IL2. In addition, purified CD56^+^ cells secreted GM-CSF under the stimulus of both classes of ODNs in collaboration with IL2. However, mixing purified CD56^+^ cells with purified PDCs reintroduced ODN specificity. CpG ODNs and IFNα inhibited the GM-CSF secretion induced by PyNTTTTGT ODNs in hPBMC, and IFNα also inhibited the ODN-induced secretion of this cytokine in purified CD56^+^ cells. These observations strongly suggest that IFNα produced by PDCs is responsible for most, if not all, the CpG ODN-mediated inhibition of the GM-CSF secretion induced by PyNTTTTGT ODNs in hPBMC. Furthermore, IMT504 inhibited the secretion of IFNα induced by CpG ODNs acting on PDCs. This inhibition seems to be the result of a specific interference in the TLR9 pathway since IMT504 is a poor inhibitor of the IFNα induced by poly-IC, an agonist of the TLR3 receptor. In addition, according to our results using HEK 293 cells to evaluate the ODN action on the TLR9 pathway, IMT504 inhibited signaling by CpG ODNs in a dose-dependent manner. Therefore, since it is well known that activation of NK cells by CpG ODNs is mainly mediated by their ability to induce IFNα secretion [29,30], we can conclude that there is a reciprocal interference between CpG ODNs and PyNTTTTGT ODNs regarding their respective patterns of CD56^+^ cell activation. This strongly suggests that synthetic CpG ODNs and PyNTTTTGT ODNs mimic independent natural stimuli that may direct the immune response toward alternative pathways. The existence of these alternative pathways has been obscured by the existence of hybrid ODNs, such as the widely studied ODN 2006, whose structure includes active elements of the two major classes of immunostimulatory ODNs for human cells. A schematic representation of the main conclusions of this study is presented in Fig. 10.

According to the state of art in the knowledge of the biology of CpG and PyNTTTTGT ODNs, it seems reasonable to speculate that synthetic CpG ODNs, which induce IFNα, should mimic some important early step during viral infection, which, as mentioned above, may well be stimulation of the internal TLR9 receptor by naked DNA viral genomes. Similarly, PyNTTTTGT ODNs should mimic the effect of another natural aggressive stimulus, which may require a different defensive response.

Regardless of which natural stimulus represents each major class of human immunoactive ODNs, it is important to discuss the biological consequences of stimulation of CD56^+^ cells by PyNTTTTGT ODNs. NK cells have been recognized as a key link between innate and acquired immune response and it has also been recognized that T cells play a critical role in NK cell activation by secreting IL2, which allows NK cells to productively respond to a number of stimuli during infection [21]. Furthermore, a central role for NKT cells in peripheral tolerance and protection against autoimmune disease, cancer and bacterial, parasitical, fungal and viral infections has been by now clearly established [31–36]. Therefore, CD56^+^ cells are emerging as key components of an important node in the circuit of homeostatic response initiated by the sensing of cellular damage that can be a consequence of infection, autoimmune attack, cancer or trauma. Thus, the strong stimulation of CD56^+^ cells by PyNTTTTGT ODNs can presumably be used for vaccine improvement and for reinforcement of the defensive homeostatic response in many pathological conditions. Several studies performed in animal models agree with this hypothesis and grants future clinical studies using PyNTTTTGT ODNs as adjuvants and therapeutic agents [12–18,37].
